# Impact of an Interactive Health Corner Using the Culinary Education Approach in Promoting Long-Term Dietary Changes among Patients Who Seek Public Primary Care Services

**DOI:** 10.3390/ijerph191811488

**Published:** 2022-09-13

**Authors:** Lynette Mei Lim Goh, Li Ming Chow, Su Yi Ng, Dana Wai Shin Chow, Raymond Boon Tar Lim

**Affiliations:** 1 Clinical Services, National University Polyclinics, National University Health System, Singapore 609606, Singapore; 2 Saw Swee Hock School of Public Health, National University of Singapore, National University Health System, Singapore 117549, Singapore

**Keywords:** cooking demonstrations, cooking intervention, healthy plate, nutrition education, food choices, healthy diet

## Abstract

An unhealthy diet is a major risk factor for chronic diseases. Although nutrition education and cooking demonstrations have resulted in favourable dietary changes, it is unclear whether this is sustainable for longer periods. This study aims to evaluate the long-term impact of a nutrition-led cooking intervention using the culinary education approach on dietary patterns based on My Healthy Plate (MHP). This was a quasi-experimental study involving patients who sought public primary care services in two polyclinics (mean age 59.3 years old). A self-administered survey was done at baseline, 6 months, and 1 year for both the intervention and the comparison groups. Participants in the intervention group were exposed to the health corner, which provided nutrition education and cooking demonstrations using the culinary education approach. A total of 216 participants completed the study at 1 year with a follow-up rate of 86%. Adjusted risk ratios (aRR) were obtained from negative binomial regression. Compared with the comparison group, participants in the intervention group were more likely to report adhering to the requirements of MHP at 6 months (aRR 1.83, 95% CI 1.12–2.99) and 1 year (aRR 1.54, 95% CI 1.10–2.16). Participants in the intervention group were less likely to add salt or sauces to food at 6 months (aRR 0.29, 95% CI 0.12–0.75) and 1 year (aRR 0.21, 95% CI 0.07–0.61) and more likely to remove fat when eating meat at 1 year (aRR 0.30, 95% CI 0.13–0.67) than the comparison group. The interventions at the health corner had a positive impact in helping patients achieve MHP recommendations, not adding salt and sauces to their food, and removing animal fat before eating. There is potential for expanding this initiative to improve healthy eating practices in other polyclinics.

## 1. Introduction

The World Health Organization (WHO) reports that non-communicable diseases (NCDs) kill 41 million people each year, equivalent to 71% of all deaths globally [1]. Other than a sedentary lifestyle, an unhealthy diet is a major risk factor for NCDs. An unhealthy diet includes excessive calories, fat, refined sugars, and salt consumption, as well as insufficient fibre. Having a healthy diet, engaging in regular physical activity, maintaining a healthy body weight, and avoiding tobacco are ways to prevent or delay the onset of intermediate metabolic risk factors such as obesity and raised blood glucose [1,2,3]. These risk factors are often the precursors to chronic diseases such as diabetes, hypertension, heart disease, and stroke.

Strategies that improve the outcome of these diseases often focus on improving unhealthy diets. Limited food preparation skills and low levels of self-efficacy towards preparing healthy meals have been reported as the main barriers to healthy eating [4,5]. Imparting nutrition knowledge through the provision of healthy eating messages and guidelines only informs people about healthy diets and healthy food choices. People still need to acquire skills on healthy food preparation [6]. The ability to plan and prepare meals ahead of time and to have greater self-efficacy in cooking abilities are key factors that can help people overcome barriers to cooking from scratch [7]. Therefore, cooking skill interventions that have the potential to improve dietary quality [8,9] are required. These interventions have been reported to help individuals make healthier food choices and thus meet nutrition guidelines [10]. In addition, the inclusion of cooking demonstrations as part of the strategy was positively correlated with fruit and vegetable intake [11]. A realist synthesis also found that nutrition education and cooking skills interventions do produce a range of positive outcomes such as increase in knowledge, cooking skills, and dietary change [12]. Furthermore, the psychosocial benefits of healthy food preparation (beyond gaining positive nutritional value) are likely to be helpful in motivating people to commit to healthy eating and to cooking healthy meals more frequently (as opposed to eating out or buying takeout) [13]. Therefore, improving cooking skills is a popular strategy in promoting healthy eating [14].

While various studies have been conducted to evaluate the effectiveness of cooking related interventions, there are still gaps in the literature. Firstly, most studies have not evaluated the impact of such interventions over a longer time frame. The follow up period of these studies typically ranged from 2 weeks to 6 months post-intervention [14,15,16]. However, a long-term change in dietary patterns was defined as having at least a 1-year follow-up from the baseline assessment based on a recent review [17]. While various studies have focused on dietary modification, relatively few have incorporated long-term follow-up or reported successful outcomes regarding maintenance of dietary behaviours [17]. For example, the impact of acquiring cooking skills on longer-term healthy eating behaviour has not yet been established [14,15,16]. In recent years, brief cooking interventions using the culinary education approach have shown to have a significant effect in increasing people’s confidence in preparing healthy meals [8,18,19]. In fact, the culinary education approach is an emerging evidence-based discipline that aims to improve eating behaviours through integrating nutritional science with food preparation. The intention is to help create positive behaviour change by not just providing knowledge but by teaching specific skills that may aid in creating lasting change [20]. While this approach has been utilised to promote healthy eating in public venues such as community cafes, this has yet to be applied in the primary care clinic setting [20]. 

Secondly, most health promotion programmes that included cooking as part of the programme were registered planned programmes, where participants had to attend a fixed number of sessions over a period of time ranging from a week to two years [8,9,16]. This might not always be feasible in the primary care or community-based setting due to time constraints and the high movement rate of people [8,9,16]. One opportunistic way of outreach is thus to target patients when they are waiting in the clinics to be seen by the primary care providers. Primary care waiting rooms could be potential sites of health promotion, where opportunities for patient education about disease prevention and treatment information for common illnesses are provided [21]. This allows the time spent waiting to be utilised efficiently to educate patients on how to improve their dietary behaviours. 

Thirdly, most studies focused on very specific aspects of the dietary behaviour such as improving fruit and vegetable intake [8]. This might not be comprehensive enough. The Plate Model, which is a method of teaching meal planning, has been increasingly used as an alternative to the traditional food exchange method for teaching portion control [22]. Intervention programmes that focused on teaching portions or healthy plate have been shown to be effective in reducing body weight, improving dietary behaviour, Type 2 diabetes, and in increasing nutrition knowledge [23,24,25,26]. This is also more holistic than focusing on increasing specific food intake. 

Similar to the rest of the world, there is also an increasing prevalence of NCD metabolic risks in Singapore. It has been forecast that the obesity prevalence will quadruple from 4.3% in 1990 to 15.9% in 2050 in Singapore [27]. If nothing is done to slow down the increasing obesity prevalence, the number of residents living with diabetes will reach one million by 2050 [27]. One major contributing factor to the metabolic risks is no doubt an unhealthy diet [28,29,30]. To encourage healthy eating, the Health Promotion Board, a statutory board under the Ministry of Health in Singapore committed to promoting healthy living, recommends the use of My Healthy Plate (MHP), which is a modification of the Plate Model. MHP consists of half a plate of vegetables and fruit, one quarter plate of protein, and one quarter plate of wholegrains. In Singapore, the first point contact of individuals with these NCD metabolic risks is the polyclinic in the primary care setting. Polyclinics are public healthcare institutions that house primary care doctors and other healthcare professionals such as nurses and dietitians to provide primary care services. Thus, the objective of this study is to evaluate whether the Health Corner (HC), which provides short nutrition education alongside cooking demonstrations using the culinary education approach, adds value as an intervention initiative to improve patients’ dietary behaviours compared with routine care and whether the changes are sustainable in the longer term. 

## 2. Materials and Methods

### 2.1. Public Primary Care Setting in Singapore and Needs Assessment

The National University Polyclinics (NUP) group is a major primary care institution in Singapore that operates 7 polyclinics. The clinics together see an annual attendance of close to 2 million, with half of the cases related to chronic diseases, making this setting an ideal venue for health promotion. A needs-assessment study showed that the prevalence of not meeting the recommendations of MHP amongst polyclinic patients was 42.7%, citing the main barriers such as lack of confidence and lack of skills in preparing or choosing healthy food, healthy food not being tasty, and healthy food being costly [4]. Our pilot study showed that nutrition education accompanied by cooking demonstrations using the culinary education approach at one polyclinic had a positive impact of delivering healthy messages to encourage healthy eating practices, and 84% of participants made positive changes after receiving the intervention at the 6-month follow-up [31]. 

### 2.2. Study Design

We used a quasi-experimental design, i.e., pre-test and post-test with a comparison group using a self-administered survey questionnaire. The intervention site was purposively selected due to the presence of the Health Corner (HC), which we named the Great Simple Tasty (GST) corner, which provides nutrition education accompanied by cooking demonstrations and food tasting sessions using the culinary education approach. No other site was able to accommodate the HC due to various logistical and operational reasons, therefore a randomised design would be infeasible. The control site was chosen where the sociodemographic profile of the patients was largely similar to that of the intervention site.

### 2.3. Participants

The inclusion criteria for participants included any patients aged at least 21 years of age who visited the polyclinic and were able to read or communicate in English or Mandarin, had not visited the health corner before, and did not have significant cognitive impairment on medical follow-up or presence of dementia. All patients were approached, of whom all eligible patients were invited to join the study. Informed consent was taken if they agreed to participate. The participants were recruited over a 6-month period from March 2019 to August 2019.

### 2.4. Sample Size Calculation

Out of those meeting the healthy plate, the anticipated increase at baseline from a previous study [4] was from 50% to 70% at the 1-year post-intervention mark in the intervention group. In order to achieve a 5% precision level and 95% confidence interval, this was compared with no change in the comparison group of a minimum sample size of 100 participants per group. Accounting for a 20% attrition rate at the 1-year follow-up, we aimed to recruit 125 participants at baseline for each group.

### 2.5. Intervention Programme and Comparison Group

The aim of the intervention was to guide people in acquiring basic cooking skills to prepare simple, tasty, and low-cost meals at home and to increase their nutritional knowledge and acceptance to adopt healthier food choices. The intervention programme was designed based on the social cognitive theory [32] and the culinary education approach [20]. Based on the social cognitive theory, this was achieved through three constructs, starting with nutrition education where the construct of behavioural capability was applied to equip participants with the knowledge and skill on healthy eating. Next, cooking demonstrations, where the construct of observational modelling was applied so that participants could watch the actions on how to prepare simple and healthy meals and, finally, food tasting sessions, where the construct of reinforcements was applied to increase the likelihood of participants adopting healthier food preparation and healthier food choices in their diet. We also applied the culinary education approach that consisted of simple nutrition education and instruction in nutritious cooking skills, including how to choose healthier food choices, meal planning, and preparation [20]. 

The intervention site was located at Bukit Batok Polyclinic in the north-west region of Singapore. The majority of the patients visited the clinic every two-to-three months for their follow-up on chronic disease. The Health Corner (HC)—the intervention site—replicates an open kitchen style café with seats where simple cooking demonstrations were conducted using induction cookers, an oven, and other common kitchen appliances. Participants would be able to observe the cooking demonstrations, taste the food prepared, and interact with the health promoter and each other with their food and drinks in a comfortable environment. The concept was developed with the purpose of having a setting that is seen to be friendly and non-intimidating in order to encourage participants to come and learn.

The HC operating hours were from 9 am to 12 noon from Monday to Friday; it was located at the chronic disease clinic waiting area and the sessions were on-going throughout the HC operating hours and did not require any prior registration. The daily sessions at the HC were conducted by a health promoter who was recruited solely to run the HC and she conducted the sessions in English and Mandarin, depending on the language preferred by the participants. The health promoter was trained by the dietitians and attended the basic food hygiene and safety course, attained certification, and was assessed to be competent by the dietitian before she could run the sessions. The sessions included education about ‘My Healthy Plate’, recipe modifications, making healthier food choices when shopping or eating out, and cooking demonstrations to show how healthy food and/or meals were prepared. The health promoter would repeat the sessions and tailor it to participants who visited accordingly, as there was no set timing since patients walk in and out throughout the day. Participants had the liberty to stay as long as they liked after the health promoter had finished going through one round of teaching about My Healthy Plate and demonstrating how to prepare the healthy meal for the day, which took about 30 to 60 min. There was a calendar of recipes planned by the dietitians for the health promoter to follow to ensure that there was a variety of different ethnic dishes and also to tie in with popular dishes prepared during festive periods. Recipes were all modified to be lower in fat, sugar, sodium, and higher in fibre. Nutritional analysis of each recipe was done using Nutritionist Pro Software version 6.1 by Axxya Systems, Washington, DC, USA. 

Participants were also able to taste the food that had been cooked and were given recipes and education materials to take home. Low-cost healthy food samples were also given to participants to take home, with the aim to encourage them to try the recipe at home. In addition, the dietitians prepared and uploaded recipe cards on the internet to provide additional recipes for patients who were keen to prepare other healthy dishes that were prepared at the HC on days when they were not in clinic. 

The control site was located at Pioneer Polyclinic, in the west region of Singapore. Participants in the comparison group were exposed to the static health promotion messages on the wall murals, floor, and staircase in the clinic. Similarly, the majority of the patients visited the clinic every 2–3 months for their follow-up on chronic disease. In addition, they were given educational materials on healthy eating to read.

### 2.6. Data Collection and Survey

Data was collected using a self-administered survey questionnaire at baseline, 6 months and 1-year post-intervention for both the intervention and the comparison groups. The informed consent form and the survey questionnaire were available in both English and Mandarin. For the intervention group, the participants were recruited and asked to fill in the survey questionnaire before the interviewer brought the patient to the health corner to attend the session at the health corner. At 6 months and 1 year from the baseline, they were contacted again to fill in the same survey questionnaire. Similarly, participants in the comparison group also filled up the survey questionnaire at recruitment. They were contacted again at 6 months and 1 year from the baseline to fill up the same survey questionnaire. Participants in the intervention group were exposed to the health corner for the first time after they were recruited and had completed the baseline survey questionnaire. As the health corner did not require registration, participants had the freedom to attend again if they wanted. 

For each participant, the interviewer was nearby to provide clarification if required. To ensure participants’ confidentiality, non-personal identifiers assigned to each individual were used on all study documents. To minimise the possibility of social desirability bias, we explained that there were no right or wrong answers and the information from the survey questionnaire will help us to improve future health promotion programmes. We also ensured that the survey questions were non-judgemental and non-sensitive so that the participants would be comfortable in providing their honest responses.

The survey included questions to study current dietary habits, including their knowledge and eating practices pertaining to MHP, barriers to fulfilling the healthy plate, attitude towards making dietary changes to improve their health, and if the heath corner and/or education materials were effective in improving understanding, motivation, confidence, and changing dietary habits. The Cronbach’s alpha coefficient of the survey was 0.72, which indicated an acceptable internal consistency.

### 2.7. Outcome Evaluation Measurements

#### 2.7.1. Primary Outcome

To assess whether participants were able to meet the requirements of MHP, we asked this question: ‘Based on your diet on a usual day, are you able to fulfil the requirements of ‘My Healthy Plate’?’ The image and description of MHP was shown and described in the survey questionnaire. Participants who responded with the options of ‘Usually or always (more than 4 times a week)’ to the question were classified as meeting the requirements of MHP. The measurement was known to patients in the polyclinics because previous assessment had been conducted in the same setting and they had indicated familiarity with the measure [4]. In addition, there were periodic public advertisements of the MHP at a national level through the various mass media channels, hence participants would be familiar with this recommendation [4]. 

#### 2.7.2. Other Outcomes

To assess whether participants had other dietary changes, we also asked questions on their dietary practices. Some of the questions were: ‘When you eat meat, do you remove the fat/skin?’; ‘At the table, do you add salt or sauces to your foods?’ 

#### 2.7.3. Process Evaluation Measurements

For the process evaluation, we included a few questions in the follow-up survey questionnaire for the intervention group. This included questions to assess the extent of: (i) the influence of the nutrition education and cooking demonstration, respectively, on their understanding to manage their health, (ii) the influence of the nutrition education and cooking demonstration, respectively, on their confidence to manage their health, and (iii) the change in their eating and cooking habits, respectively, after receiving the intervention.

### 2.8. Statistical Analysis

The baseline equivalence of sociodemographic characteristics and the primary outcome in the intervention and comparison groups were compared using χ2 test for categorical and independent sample *t*-test for continuous variables. Before we carried out the independent sample *t*-test, we applied the Shapiro–Wilk Test and plotted the histogram to check the normality of the data. The continuous variables such as age showed a normal distribution before we proceeded with the test. Any statistically significant variable between the comparison and the intervention group in terms of sociodemographic characteristics and the baseline primary outcome would be adjusted in the outcome evaluation. The proportion of participants with the behavioural outcome was calculated at baseline (those who completed follow-up only) and at follow-up. The negative binomial regression was applied to generate both crude risk ratio (RR) and adjusted risk ratio (aRR) of the behavioural outcomes in the intervention versus the comparison group at follow-up. For the behavioural outcome, an RR above 1 indicated more participants reporting that outcome in the intervention than the comparison group at follow-up. The inverse was true when the RR was below 1. Missing data were negligible since they ranged from 0.8% to 5.9% across all the data fields. Complete case analyses were carried out, where we utilised only the cases in the data set for which there were no missing values on any of the key variables. Therefore, for the 6-month and 12-month follow-up analyses, we used the final numbers remaining at 12 months. All analyses were performed using SPSS 26.0.

## 3. Results

**Figure 1:**
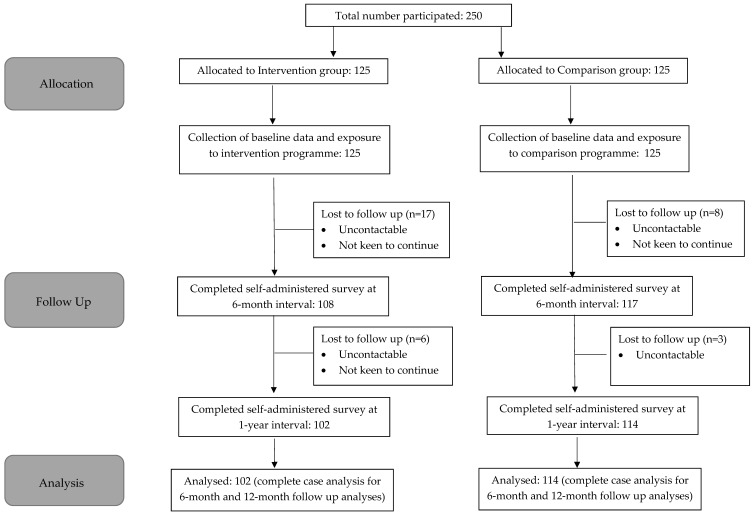
Consort Flow Diagram.

A total of 250 participants were recruited at baseline, with an equal number of participants recruited at the intervention site (*n* = 125) and control site (*n* = 125). At the 6-month follow-up, a total of 225 participants continued the study, with 108 participants from the intervention group and 117 participants from the comparison group. A total of 25 participants dropped out due to time constraints, or they were uncontactable. The number of participants who completed the study at the 1-year follow up was 216, with 102 participants from the intervention group and 114 participants from the comparison group, with a follow-up rate of 86%. Participants dropped out of the study because they were uncontactable or uninterested to continue in the study due to time constraints (Figure 1).

There was no significant difference between those who completed the 1-year follow-up and those who dropped out in terms of sociodemographic characteristics such as age, sex, and ethnicity in both the intervention and the comparison groups.

Table 1 shows the comparison of sociodemographic characteristics and the primary outcome for the intervention and the comparison groups at baseline. Participants in both groups had similar distributions in marital status and education level. The intervention group had a greater proportion of females and a higher mean age compared with the comparison group. There was also significant difference in ethnicity. At baseline, there was no difference in the primary outcome measurement of My Healthy Plate.

Table 2 shows the crude and aRR of the dietary behaviours of the intervention and comparison groups at the 6-month follow-up. The aRR were obtained after adjustment for age, sex, and ethnicity, since these sociodemographic characteristics were different between the intervention and the comparison group. Comparing with the comparison group, participants in the intervention group were more likely to report adhering to the requirements of MHP at the 6-month follow-up (aRR 1.83, 95% CI 1.12–2.99). Participants in the intervention group were less likely to add salt or sauces to food at 6 months (aRR 0.29, 95% CI 0.12–0.75) than the comparison group.

Table 3 shows the crude and aRR of the dietary behaviours of the intervention and comparison groups at 1-year follow-up. Participants in the intervention group were more likely to adhere to the requirements of MHP at 1-year (aRR 1.54, 95% CI 1.10–2.16) compared with the comparison group. Participants in the intervention group were less likely to add salt or sauces to food at 1 year (aRR 0.21, 95% CI 0.07–0.61) and more likely to remove fat when eating meat at 1 year (aRR 0.30, 95% CI 0.13–0.67) than the comparison group.

**Figure 2:**
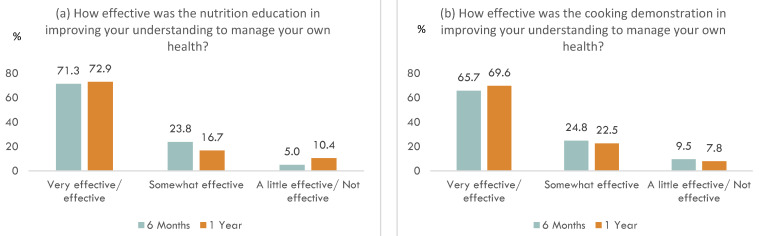

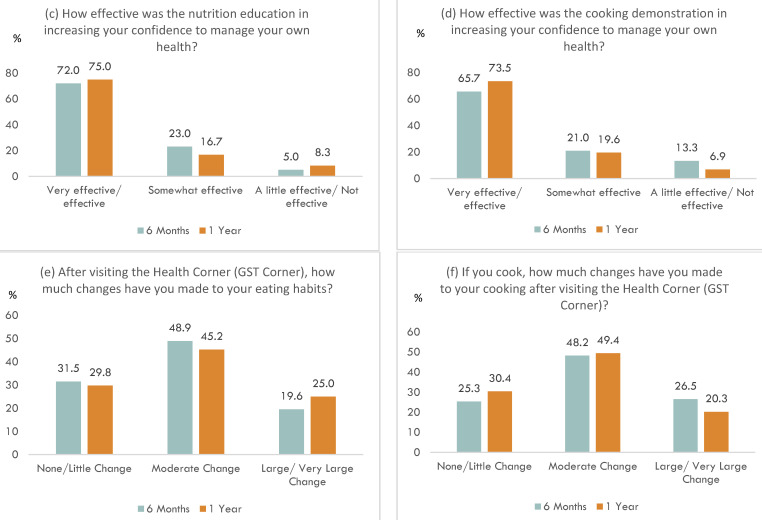
Results of the process evaluation in the intervention group; (**a**) Effectiveness of the nutrition education in improving understanding to manage own health, (**b**) Effectiveness of the cooking demonstration in improving understanding to manage own health, (**c**) Effectiveness of the nutrition education in increasing confidence to manage own health, (**d**) Effectiveness of the cooking demonstration in increasing confidence to manage own health, (**e**) Changes to eating habits after visiting the Health Corner, (**f**) Changes to cooking habits after visiting the Health Corner.

Figure 2 shows the results of the process evaluation in the intervention group. At both the 6-month and 1-year follow-up, more than 70% of the participants in the intervention group reported that the nutrition education was effective/very effective in improving their understanding to manage their own health. This was similar for the case of the cooking demonstration, where the proportion was more than 65% at the two follow-up time points. For confidence to manage their health, more than 70% of the participants in the intervention group reported that the nutrition education was effective/very effective in helping them to achieve that. This was similar for the case of the cooking demonstration, where the proportion was more than 65% at the two follow-up time points. At both the 6-month and 1-year follow-up, more than two-thirds of the participants in the intervention group reported at least a moderate change in their eating habits. This was similar for the case of the cooking habits.

## 4. Discussion

The results of the study showed that the participants in the intervention group were more likely to report adhering to the requirements of My Healthy Plate (MHP) at both the 6-month and 1-year follow-up compared with the comparison group. So far, to the best of our knowledge, no studies have been done to explore the impact of a behavioural intervention on the provision of nutrition education alongside cooking demonstrations using the culinary education approach in a primary care setting. 

Our intervention programme was an adaption of the Teaching Kitchens and culinary medicine, which is growing in popularity and effectiveness [33]. The findings echoed other studies in which those participating in hands-on kitchen-based nutrition education were more likely to follow a healthier dietary pattern in New Orleans [34], as well as those participating in a community culinary coaching programme were more likely to report greater satisfaction in a healthier diet in Israel [35]. Culinary medicine is an emerging discipline in clinical and public health education that provides healthcare professionals and community members with food-based knowledge and skills. With the hands-on teaching of kitchen education to individuals, culinary medicine provides eaters with tangible strategies for incorporating healthier and tastier food in their lives. Our study’s findings showed that this novel interactive approach to culinary and nutrition education could also take place in primary care clinics, hence providing another venue for health promotion to take place. 

My Healthy Plate was used as the main teaching curriculum as it is a simple tool that emphasises all the important food groups for building healthy meals. It is also a simple method of teaching meal planning that enhances the connection between dietary theory and practice, focuses on total dietary quality, is easy to remember and understand visually, and all cuisines can be incorporated into the model [22]. As knowledge is a building block toward behaviour change [24] and cooking skills are related to food choices [10], we hoped that with time, increased nutrition knowledge and showing simple ways of preparing healthy food may lead to improvements in the desire and ability to prepare healthy and well-balanced meals. Barriers for not meeting the MHP recommendations in our needs assessment prior to this intervention study were similar to previous studies, where participants reported not meeting recommendations because they perceived healthy food as not tasty, had a lack of self-motivation to have a healthy and balanced diet, had a lack of knowledge on how to have a healthy and balanced diet, and had a lack of skills on how to prepare healthier home-cooked food [4,5]. Keeping in mind that these were common obstacles to having a healthy diet, the health corner was named the ‘GST Corner’ from the start, which means ‘Great Simple Tasty’, and our intervention focused on cooking simple meals that were tasty.

The programme at the health corner aimed to promote confidence, to teach patients how to prepare simple, tasty, and budget meals in the hope that participants are empowered to prepare healthy meals with their limited resources and to overcome the perception that healthy meals are expensive to prepare. When patients have the confidence to take action and overcome barriers, they are more motivated to take small steps to change behaviours such as dietary habits. Through nutrition education, observation from the cooking demonstrations and the reinforcement after tasting the healthy food prepared, they may change their attitude and beliefs about healthy food, develop skills to replicate what they have seen, and in turn experience a result in behaviour change. Other positive reinforcements include providing recipes, education materials, and food samples to cook at home to help reinforce the learning. In addition, siting the health corner at a prominent location allowed easy and continual access. However, we recognise that this alone does not change environmental constraints such as living costs and food affordability, which are also key factors in sustaining long-term behavioural changes. 

Apart from MHP, the intervention also aimed to assess whether participants had other dietary changes such as improvements in fat and sodium intake, both of which contribute to NCD metabolic risks. High salt intake raises blood pressure and this increases the risk of stroke and cardiovascular diseases. Nutrition education that includes food preparation demonstrations has been found to improve blood pressure, reduce sodium intake, and improve dietary behaviours for maintaining a low-salt diet [36]. Another study that incorporated a ‘cook and eat’ component in their intervention to encourage lower fat intake found that the interventions with the cooking skills element had greater reductions in energy density than the intervention without the cooking element [37]. In our study, participants in the intervention group were less likely to add salt or sauces to their food at 6 months and this was sustained at a 1-year follow-up. In addition, the intervention group was also more likely to remove fat when eating meat at the 1-year follow-up. This shows that, other than learning about healthy plate, other healthy behaviours such as salt and fat intake had improved.

This study has some limitations. There may be social desirability bias as the information was self-reported. In addition, we did not use the food frequency questionnaire and the 24-h dietary recall because this was a pragmatic intervention study in the busy polyclinic setting and we wanted to reduce the question burden on participants. We also did not measure the dosage response to find out whether those who visited the health corner more frequently on their own resulted in better results. In addition, because randomisation was not used, this limited the study’s ability to conclude a causal association between an intervention and an outcome. That said, we have reduced this bias by choosing a comparison site that was largely similar to the patient profile in the intervention site, as well as adjusting for any sociodemographic differences between the two groups during data analysis. Another limitation was the possibility of residual confounding between the comparison and the intervention group. We have adjusted for sociodemographic differences including sex (the biggest difference), and there were no significant differences in dietary habits at baseline between both sexes. However, there could still be differences in cooking frequency, since this was not assessed in the survey questionnaire. In addition, the generalisability of the study would be limited to individuals who sought public primary care services. Despite the limitations, there were various strengths. Our study was the first to report findings from the participation in a nutrition education with cooking demonstrations using the culinary education approach in a primary care setting. The results were promising as they showed a sustainable and improved healthy dietary behavioural change at the 1-year follow-up. Another strength would be the change in programme development from focusing on specific components of a healthy diet, such as eating less fat or sugar, to a more holistic approach of using the My Healthy Plate to improve healthy eating and cooking practices. 

## 5. Conclusions

Primary prevention is the most effective and affordable way to prevent chronic disease and dietary management positively impacts health outcomes across the lifespan [38].

The findings in this study showed that incorporating nutrition education and cooking demonstrations using the culinary education approach at the health corner had a positive impact of helping patients improve their diet at the 6-month follow-up and this was sustained at 1-year. There is potential for expanding this initiative to improve healthy eating practices in other polyclinics and healthcare settings to help prevent and manage diet-related chronic diseases.

## Figures and Tables

**Table 1:** Comparison of sociodemographic characteristics and the primary outcome for intervention and comparison groups at baseline.

Characteristic		Comparison (*n* = 125)	Intervention (*n* = 125)	*p*-Value
Sex	Male	56 (44.8)	22 (17.6)	<0.001 *
	Female	69 (55.2)	103 (82.4)	
Age	Age, mean (SD)	57.6 (9.7)	61.0 (9.7)	0.006 *
Ethnicity	Chinese	75 (60.0)	101 (80.8)	0.005 *
	Malay	33 (26.4)	16 (12.8)	
	Indian	15 (12.0)	7 (5.6)	
	Other	2 (1.6)	1 (0.8)	
Marital Status	Currently single	21 (16.8)	31 (24.8)	0.120
	Married	104 (83.2)	94 (75.2)	
Education ^+^	No formal education/primary	32 (25.6)	31 (25.0)	0.980
	Secondary	53 (42.4)	53 (42.7)	
	Junior College/Institute of Technical Education/Polytechnic	24 (19.2)	22 (17.7)	
	University and beyond	16 (12.8)	18 (14.5)	
Primary outcome	I have not or seldom been doing ‘My Healthy Plate’	76 (60.8)	84 (67.2)	0.292
	I have been doing ‘My Healthy Plate’ most of the time	49 (39.2)	41 (32.8)	

^+^ One of the questionnaires contained a blank response on educational level for the intervention group therefore there was no information on the educational level pertaining to one of the participants in the intervention group. * significant level.

**Table 2:** Crude and adjusted risk ratio of dietary behaviours of the intervention and comparison groups at 6-month follow-up.

Dietary Behaviour	Comparison (*n* = 114) N (%)	Intervention (*n* = 102) N (%)	Crude RR	*p*-Value	Adjusted RR ^+^	*p*-Value
Adhering to the requirements of MHP most of the time * (Not adhering as the reference group)	31 (27.9%)	48 (50.0%)	1.80 (1.23–2.63)	0.003	1.83 * (1.12–2.99)	0.015 *
Frequency of deep-fried foods at more than 3× a week/ daily (None/monthly/1–3× a week as the reference group)	20 (17.5%)	6 (5.9%)	0.33 (0.14–0.78)	0.012	0.54 (0.19–1.55)	0.253
Do not remove fat among those who eat meat (Remove as the reference group)	20 (17.5%)	8 (7.8%)	0.49 (0.23–1.03)	0.058	0.59 (0.27–1.28)	0.182
Do not remove fat among those who eat poultry (Remove as the reference group)	21 (18.4%)	10 (9.8%)	0.51 (0.25–1.04)	0.063	No Model Convergence	
Usually add salt or sauces to their foods (Usually do not add as the reference group)	24 (21.1%)	5 (4.9%)	0.23 (0.09–0.57)	0.002	0.29 * (0.12–0.75)	0.010 *

^+^ adjusted for age, sex, and ethnicity; * The number of responses for the comparison and intervention group was 111 and 96, respectively, for this measure.

**Table 3:** Crude and adjusted risk ratio of dietary behaviours of the intervention and comparison groups at 1-year follow-up.

Dietary Behaviour	Comparison (*n* = 114) N (%)	Intervention (*n* = 102) N (%)	Crude RR	*p*-Value	Adjusted RR ^+^	*p*-Value
Adhering to the requirements of MHP most of the time * (Not adhering as the reference group)	42 (37.8%)	56 (58.3%)	1.54 (1.15–2.07)	0.004	1.54 * (1.10–2.16)	0.013 *
Frequency of deep-fried foods at more than 3× a week/ daily (None/monthly/1–3× a week as the reference group)	20 (17.5%)	4 (3.9%)	0.22 (0.08–0.63)	0.005	No Model Convergence	
Do not remove fat among those who eat meat (Remove as the reference group)	30 (26.3%)	7 (6.9%)	0.27 (0.12–0.58)	0.001	0.30 * (0.13–0.67)	0.004 *
Do not remove fat among those who eat poultry (Remove as the reference group)	32 (28.1%)	11 (10.8%)	0.39 (0.21–0.73)	0.003	No Model Convergence	
Usually add salt or sauces to their foods (Usually do not add as the reference group)	26 (22.8%)	4 (3.9%)	0.17 (0.06–0.48)	0.001	0.21 * (0.07–0.61)	0.004 *

^+^ adjusted for age, sex, and ethnicity; * The number of responses for the comparison and intervention groups was 111 and 96, respectively, for this measure.

## Data Availability

Not applicable.
